# Supplementary material for: Condensin controls cellular RNA levels through the accurate segregation of chromosomes instead of directly regulating transcription
Source: eLife. 2018 Sep 19;7:e38517. doi: 10.7554/eLife.38517 (PMC6173581; doi:10.7554/eLife.38517)
Supplement: Supplementary File 1. [file elife-38517-supp1.docx]

**Supplementary File 1. RNA levels of RNA-exosome and TRAMP components in *cut14-208* mutant cells**

|  |  |  | *cut14-208* vs wt | |
| --- | --- | --- | --- | --- |
| Protein | Systematic gene name | Protein Complex | P value adj | Log2FC |
| Rrp6 | SPAC1F3.01 | RNA-exosome | 1 | -0,24371 |
| Dis3 | SPBC26H8.10 | RNA-exosome | 1 | -0,13197 |
| Rrp41 | SPAC3G9.10c | RNA-exosome | 1 | -0,23751 |
| Rrp43 | SPBC17D1.03c | RNA-exosome | 1 | -0,10895 |
| Cti1/Rrp47 | SPCC1739.07 | RNA-exosome | 1 | 0,00185 |
| Csl4 | SPCC1840.11 | RNA-exosome | 1 | -0,13319 |
| Rrp4 | SPAC2F7.14c | RNA-exosome | 1 | -0,18844 |
| Rrp40 | SPAC22A12.12c | RNA-exosome | 1 | 0,02820 |
| Rrp46 | SPBC115.01c | RNA-exosome | 1 | -0,16979 |
| Mtr3 | SPBC211.08c | RNA-exosome | 1 | 0,03692 |
| Rrp42 | SPBC16G5.10 | RNA-exosome | 1 | -0,01230 |
| Rrp45 | SPCC757.08 | RNA-exosome | 1 | -0,09132 |
| cid14 | SPAC12G12.13c | TRAMP | 1 | -0,17385 |
| Air1 | SPBP35G2.08c | TRAMP | 1 | -0,32050 |
| Mtr4 | SPAC6F12.16c | TRAMP | 1 | -0,02359 |
| RNA helicase | SPAC17H9.02 | TRAMP | 1 | -0,22135 |
