# Supplementary material for: Condensin controls cellular RNA levels through the accurate segregation of chromosomes instead of directly regulating transcription
Source: eLife. 2018 Sep 19;7:e38517. doi: 10.7554/eLife.38517 (PMC6173581; doi:10.7554/eLife.38517)
Supplement: Supplementary File 3. [file elife-38517-supp3.docx]

**Supplementary File 3.** Primers and RNA-FISH probes used in this study

| primer | Left | Right | experiment |
| --- | --- | --- | --- |
| ncRNA.540 5’ | ttgcagtaatgctcacggtag | gttgtgaaaactctccgtctcc | ChIP, RT-qPCR |
| ncRNA.540 3’ | gagaaaagatggggaaactgc | gaagacggcagaatggaaag | ChIP |
| ncRNA.489 5’ | tttatctggatggggtttatcg | gtgactcgtatgccagaagaag | ChIP |
| ncRNA.489 3’ | gccttttgttgccttctatgc | tgcttgtatgtttcggcctac | ChIP, RT-qPCR |
| mug93as 5’ | AACGGCAGTATTGGTGTGC | CGGATACACCAAGCAATGAAG | ChIP, RT-qPCR |
| mug93as 3’ | TCCACCAAGTTTACGTTCTCC | TCCACCAAGTTTACGTTCTCC | ChIP |
| act1 | gaatggatccaccaatccag | ccgatcgtatgcaaaaggag | RT-qPCR |
| nda2 | GCGTCCAACCTATGAAAACC | CCAGCAAATCGAAGAGAAGC | RT-qPCR |
| lsd90 | TTCTTCGGATAGCGACAAGG | ATACCCAAGGCATGACGAAC | ChIP, RT-qPCR |
| spac23h3.15c | GTTACAGCGGTGAGGGATATG | TGCAGTTGTACGAGCATTACG | ChIP, RT-qPCR |
| spbc660.05 | TCCAGGCTTGCATATTCCTC | AAGAACACTGCCGGCTAAAG | ChIP, RT-qPCR |
| cendH1 | cgctttgttgtcgtggacta | aacacggcgataagaaatgg | ChIP |
| NTS1 | GGCTGGATCTGGCAAAAAG | TCCAGCCTTCTCCATTTCAC | ChIP |
| CAR1806 | ATTGTCAACACGACCCCAAG | GGATGAAGTCGGTGCTTTTG | ChIP |
| CAR810 | TTCTTCACTGTTGGGCAAGC | CTACGTTGGCAATCTGAACG | ChIP |
| CAR928 | TTCGAACCTGGAGAAGAACG | GCCGTTGAAACTTTTTGTCG | ChIP |
| hsp9 | GTCAAAACGTGTTTTCGCTTATG | TGTAAACAATCGGCATTGAGAG | ChIP |
| ymr31 | GCAAGCTGTTGAATCTGGTG | AGTAATGGGATGCGTGATCG | ChIP |
| atf1 | TCAAGTTAAAATTTGCACCTTCAC | ACATCCTCAATGTAGCGACAAAC | ChIP |
| kgd1 | ATTGCGACGCGATGTTTG | TGCACACTCTCAACCAGAGG | ChIP |
| SPCC16A11.15c | TGAATTTTGGGTTTTCGACAG | AGGTTGGCCCATCATCAC | ChIP |
| SPCC11E10.01 | AAACGTGAGAAACCCTGTTTG | CCGCTGTTGTGTGTATTTCC | ChIP |
| SER | TGTCCGAGTGGTTAAGGAGTTAG | TCACCAGCAGGATTTGAACC | RT-qPCR |
| SER+MET | AGGTTCAAATCCTGCTGGTG | AGGTTCAAATCCTGCTGGTG | RT-qPCR |
| LEU | AGGGTGTCCGAGTGGTTATG | AAAGGGGTGGGACTCGAAC | RT-qPCR |
| THR | CCCCTATGGCTTAGTGGTACAG | CCCCACTCGGAATCGAAC | RT-qPCR |
| TRP | GCCCCTTAACTCAGTTGGTAGAG | TGACCCCTAAGTGACTTGAACAC | RT-qPCR |
| rDNA | TTTCTGCCTTTTTCGGTGAC | TGGCATGGATTTCCCTTTAG | qPCR |
| cct2_1 | ttggatgccactttcgttaa | n.a. | smRNA-FISH |
| cct2_2 | gaaagacgggcattttcacc | n.a. | smRNA-FISH |
| cct2_3 | cagcaattgcacccacaaac | n.a. | smRNA-FISH |
| cct2_4 | agtgctttttaccaaatcgc | n.a. | smRNA-FISH |
| cct2_5 | aattttgtccattcccttag | n.a. | smRNA-FISH |
| cct2_6 | gtcacaacaatatcacccga | n.a. | smRNA-FISH |
| cct2_7 | aagaatggtagcaccgtcat | n.a. | smRNA-FISH |
| cct2_8 | gctgcattatccaaagctat | n.a. | smRNA-FISH |
| cct2_9 | ccaacttcatcatcttggac | n.a. | smRNA-FISH |
| cct2_10 | gacacacacactggtagtac | n.a. | smRNA-FISH |
| cct2_11 | gacgaagaagttcagcagcg | n.a. | smRNA-FISH |
| cct2_12 | gcattgaccataatttctgc | n.a. | smRNA-FISH |
| cct2_13 | cgaagagcatcgatagcagt | n.a. | smRNA-FISH |
| cct2_14 | tattctcgaggtcactacga | n.a. | smRNA-FISH |
| cct2_15 | gaagacagagtagttctggc | n.a. | smRNA-FISH |
| cct2_16 | ccagctgagcaaaatggttt | n.a. | smRNA-FISH |
| cct2_17 | caagcgaagcactgcatcaa | n.a. | smRNA-FISH |
| cct2_18 | tgttatccaagttcgtgcta | n.a. | smRNA-FISH |
| cct2_19 | gtttaccaccaaggatctta | n.a. | smRNA-FISH |
| cct2_20 | aatccctcatcaaggaagga | n.a. | smRNA-FISH |
| cct2_21 | ctttgggacagttaacacca | n.a. | smRNA-FISH |
| cct2_22 | ccatagccgtattagcaatt | n.a. | smRNA-FISH |
| cct2_23 | ccctagcaccaaaaactttc | n.a. | smRNA-FISH |
| cct2_24 | gttcaagttcggcaagcttg | n.a. | smRNA-FISH |
| cct2_25 | actttagccttcattttctc | n.a. | smRNA-FISH |
| cct2_26 | gctctggccaattgtaaatg | n.a. | smRNA-FISH |
| cct2_27 | aataccagcgtcagcgaaca | n.a. | smRNA-FISH |
| cct2_28 | agtctgcatgttcaatggac | n.a. | smRNA-FISH |
| cct2_29 | caaagacagccgctcaattc | n.a. | smRNA-FISH |
| cct2_30 | ttgaagcaatttcaccacca | n.a. | smRNA-FISH |
| cct2_31 | tgacaagttcaggatggtca | n.a. | smRNA-FISH |
| cct2_32 | ttcaccgataatgatctcct | n.a. | smRNA-FISH |
| cct2_33 | ggcttcaacgccagaaaact | n.a. | smRNA-FISH |
| cct2_34 | aaacaatggtgcaggcttct | n.a. | smRNA-FISH |
| cct2_35 | taactgatgagtggcaccac | n.a. | smRNA-FISH |
| cct2_36 | tcatgaatggctcgttcaga | n.a. | smRNA-FISH |
| cct2_37 | cagcgactgtttgggaaaga | n.a. | smRNA-FISH |
| cct2_38 | tcctcctaaagtgactcgag | n.a. | smRNA-FISH |
| cct2_39 | ccattagcatttcagcacaa | n.a. | smRNA-FISH |
| cct2_40 | cagaaacagcaactgccttc | n.a. | smRNA-FISH |
| cct2_41 | gtcaaagccagcattatcag | n.a. | smRNA-FISH |
| cct2_42 | aactgagctactagctcact | n.a. | smRNA-FISH |
| cct2_43 | gttgccatcataatgagctg | n.a. | smRNA-FISH |
| cct2_44 | ccatatcaagacccattgta | n.a. | smRNA-FISH |
| cct2_45 | atatcagcaatctctccttc | n.a. | smRNA-FISH |
| cct2_46 | ttcaatgcctcgagtatacc | n.a. | smRNA-FISH |
| cct2_47 | aagttgagcaccttcagatc | n.a. | smRNA-FISH |
| cct2_48 | ggtgcagcctttaatatagt | n.a. | smRNA-FISH |
| mug93as_1 | tttatcggtatggcaaggtt | n.a. | smRNA-FISH |
| mug93as_2 | gcaacccttttctcataatc | n.a. | smRNA-FISH |
| mug93as_3 | acagatacacagccaacgtg | n.a. | smRNA-FISH |
| mug93as_4 | tgccatgaatcgcatattca | n.a. | smRNA-FISH |
| mug93as_5 | aatactgccgtttctcatac | n.a. | smRNA-FISH |
| mug93as_6 | agacagttttgtgttgcaca | n.a. | smRNA-FISH |
| mug93as_7 | tacacaagtattgtccgtca | n.a. | smRNA-FISH |
| mug93as_8 | atgaagacagtcatcgaccc | n.a. | smRNA-FISH |
| mug93as_9 | tgtttgccattttgtttacc | n.a. | smRNA-FISH |
| mug93as_10 | aacctcttattttcagtgct | n.a. | smRNA-FISH |
| mug93as_11 | aggcattaataaccgtgtgt | n.a. | smRNA-FISH |
| mug93as_12 | ccatcgatgcaatcatttgg | n.a. | smRNA-FISH |
| mug93as_13 | gtatgttaagtcagtccaga | n.a. | smRNA-FISH |
| mug93as_14 | tcccaatcaattgctacaca | n.a. | smRNA-FISH |
| mug93as_15 | ggcatgtttccaaaaaaccc | n.a. | smRNA-FISH |
| mug93as_16 | gcagctcaaattcttttgca | n.a. | smRNA-FISH |
| mug93as_17 | ttcagttttgtcgtgcaaca | n.a. | smRNA-FISH |
| mug93as_18 | caagcaatggtccgacaagg | n.a. | smRNA-FISH |
| mug93as_19 | tcggattgtgttcaaggttt | n.a. | smRNA-FISH |
| mug93as_20 | atggaatgtcggttgagcag | n.a. | smRNA-FISH |
| mug93as_21 | tttcatttcctatttccaca | n.a. | smRNA-FISH |
| mug93as_22 | cccctgtttgtacattgaaa | n.a. | smRNA-FISH |
| mug93as_23 | gtacagatccaattacccat | n.a. | smRNA-FISH |
| mug93as_24 | agaacactctctattggtcc | n.a. | smRNA-FISH |
| mug93as_25 | agctcggaggacgaattaga | n.a. | smRNA-FISH |
| mug93as_26 | gataccgaaaatcactcgcc | n.a. | smRNA-FISH |
| mug93as_27 | tttcagcaatgcggtctatg | n.a. | smRNA-FISH |
| mug93as_28 | tgtggacagcaatgcgaact | n.a. | smRNA-FISH |
| mug93as_29 | aagtgcacgacaattgccaa | n.a. | smRNA-FISH |
| mug93as_30 | aattcgtggcaagatctggt | n.a. | smRNA-FISH |
| mug93as_31 | gcaattacctgtaggattgc | n.a. | smRNA-FISH |
| mug93as_32 | agacaccttagagttgcttc | n.a. | smRNA-FISH |
| mug93as_33 | atgtgcagaaacatggcagt | n.a. | smRNA-FISH |
| mug93as_34 | tcacttgaactggacccaaa | n.a. | smRNA-FISH |
| mug93as_35 | acttagccaatcgtgattgg | n.a. | smRNA-FISH |
| mug93as_36 | tatttgcgacttggacatcc | n.a. | smRNA-FISH |
| mug93as_37 | gtgatatggaggagaggact | n.a. | smRNA-FISH |
| mug93as_38 | ctctagtcatcaatccgtat | n.a. | smRNA-FISH |
| mug93as_39 | acttattttgcatgcagcta | n.a. | smRNA-FISH |
| mug93as_40 | aacatcgtctgaacctttgc | n.a. | smRNA-FISH |
| mug93as_41 | aggttcagagaatcctttgg | n.a. | smRNA-FISH |
| mug93as_42 | ctcattaactcatttgcgca | n.a. | smRNA-FISH |
| mug93as_43 | aacaccattacaccggttta | n.a. | smRNA-FISH |
| mug93as_44 | tgtaacgtttctaaccctca | n.a. | smRNA-FISH |
| mug93as_45 | cactagaggtatgctctact | n.a. | smRNA-FISH |
| mug93as_46 | ccatctgatttgaaccatct | n.a. | smRNA-FISH |
| mug93as_47 | tccaatattgttctaccaca | n.a. | smRNA-FISH |
| mug93as_48 | cacatggttatgatatgcct | n.a. | smRNA-FISH |

n.a. : not applicable
