# Supplementary material for: Condensin controls cellular RNA levels through the accurate segregation of chromosomes instead of directly regulating transcription
Source: eLife. 2018 Sep 19;7:e38517. doi: 10.7554/eLife.38517 (PMC6173581; doi:10.7554/eLife.38517)
Supplement: Supplementary File 4. [file elife-38517-supp4.docx]

**Supplementary File 4. Antibodies used in this study**

| Application | Antibody |
| --- | --- |
| IF -α tubulin | mouse monoclonal Tat1, Keith Gull |
| IF Rrp6-MYC | mouse monoclonal #9E10 Thermo Fisher |
| IF Dis3-HA | mouse monoclonal #12CA5 Sigma-Aldrich |
| IF Gar2-GFP ; ChIP Psm3-GFP | rabbit polyclonal #A11122, Life Technologies |
| ChIP RNA Pol II (ser2P) | rabbit polyclonal #ab5095 ChIP-grade, abcam |
| ChIP anti-HA | Monoclonal 16B12 (anti-HA.11, Covance) |
| ChIP anti-PK | Monoclonal anti-PK (V5) Abd Serotec MCA1360 |
| Chromosome spreads anti-Brn1-HA_6_ | Monoclonal 16B12 (anti-HA.11, Covance) |
